# Two causes of palpitations, detected by photoplethysmography on a mobile phone

**DOI:** 10.1007/s12471-018-1209-y

**Published:** 2018-11-27

**Authors:** W. Gielen, M. Gielen

**Affiliations:** Silkeborg Regional Hospital, Silkeborg, Denmark

## Answer

During the 6th (and most recent) Consensus Conference of the Atrial Fibrillation Network (AFNET) and the European Heart Rhythm Association (EHRA) advanced technologies as photoplethysmography (PPG) were mentioned for the detection of atrial fibrillation (AF). PPG has here a sensitivity and specificity of 97–100% and 92–94%, respectively, for the detection of AF compared with 12-lead ECG interpretation [1].

This puzzle shows us, though, that there is more to consider on a PPG than AF alone.

**Fig. 1:**
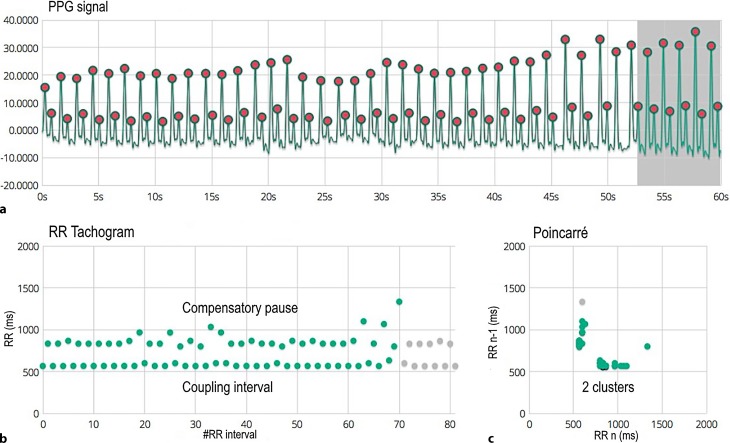
Photoplethysmogram (PPG) recording. **a** 60 s PPG signal measured at the patient’s fingertip with the phone camera. **b** RR tachogram which represents the distance between the RR intervals in milliseconds. **c** The Poincaré plot shows how the present RR interval is related to the previous one

**Fig. 2:**
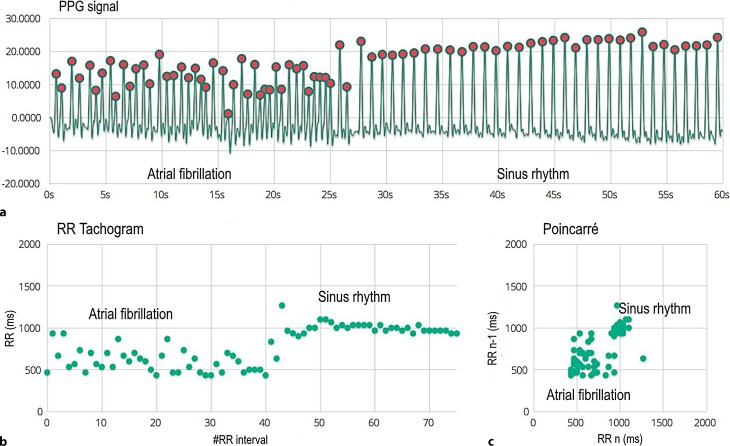
Photoplethysmogram (PPG) recording. **a** 60 s PPG signal measured at the patient’s fingertip with the phone camera. **b** RR tachogram which represents the distance between the RR intervals in milliseconds. **c** The Poincaré plot shows how the present RR interval is related to the previous one

We can observe premature extrasystoles in bigeminy in the first episode (Fig. 1). Here, panel a shows each beat followed by another beat with a short coupling interval and a lower amplitude. The tachogram (b) shows two separate lines, where the coupling interval is represented in the bottom line, and the compensatory pause in the upper line. The Poincaré plot (c) shows two separate clusters. Fig. 2 shows an episode with AF followed by sinus rhythm. Panel a initially shows an irregular faster rhythm with different amplitudes, followed by a much slower regular rhythm, the tachogram (b) shows a chaotic spread in RR intervals which stabilises afterwards when sinus rhythm is achieved, the Poincaré plot shows sinus rhythm as a condensed cluster in the middle with AF as a chaotic pattern more southwest. Afterwards, AF was also documented on Holter monitoring and the appropriate treatment was initiated.

According to this recent consensus for integrating new approaches to AF management, further investigation is needed as ECG-diagnosed AF is still the preferred method to decide on further management [1, 2]. Using these new technologies enables us to track down patients’ symptoms more conveniently and over a longer period of time, and to evaluate further with ECG if needed.
